# Multilevel Self-Management in Nursing Research: An Approach to Decrease Health Disparities in Chronic Diseases*

**DOI:** 10.17533/udea.iee.v41n2e10

**Published:** 2023-08-24

**Authors:** Evelyn Iriarte, Rosina Cianelli, Madeline Fernandez-Pineda

**Affiliations:** 1 Ph.D, MSN, RN. Adjunct Instructor at the School of Nursing, Pontificia Universidad Catolica de Chile, Santiago, Chile, Postdoctoral Fellow at the University of Colorado College of Nursing, Aurora, Colorado, U.S., and Young Researcher at Millennium Institute for Care Research, MICARE, Santiago, Chile. Email: esiriart@uc.cl. https://orcid.org/0000-0002-9618-7898 Pontificia Universidad Católica de Chile School of Nursing Pontificia Universidad Catolica de Chile Santiago Chile esiriart@uc.cl; 2 Ph.D, MPH, RN, IBCLC, FAAN. Professor of Nursing at the School of Nursing and Health Studies, University of Miami, Coral Gables, Florida, U.S. Email: rcianelli@miami.edu https://orcid.org/0000-0002-4294-5569 University of Miami School of Nursing and Health Studies University of Miami Coral Gables Florida USA rcianelli@miami.edu; 3 Ph.D, MSN, APRN, WHNP-BC. Assistant Professor at the East Carolina University College of Nursing, Greenville, North Carolina, U.S. Email: fernandezm21@ecu.edu https://orcid.org/0000-0002-9545-9484 East Carolina University East Carolina University College of Nursing Greenville North Carolina USA fernandezm21@ecu.edu

**Keywords:** chronic disease, self-care, health status disparities, healthcare disparities, nursing research, enfermedad crónica, autocuidado, disparidades en el estado de salud, disparidades en atención de salud, investigación en enfermería, doença crônica, autocuidado, disparidades nos níveis de saúde, disparidades em assistência à saúde, pesquisa em enfermagem

## Abstract

**Objective:**

To discuss multilevel self-management intervention research in nursing to decrease health disparities among people living with chronic diseases.

**Content synthesis:**

Multilevel interventions have become the core of nursing research in the last decade. However, a critical limitation of existing interventions targeting health disparities among those living with chronic diseases is the tendency to address single or individual-level factors solely.

**Conclusions:**

Nursing research is creating knowledge that may be translated into clinical practice and promoting evidence-based and innovative self-management practices to decrease health disparities and promote health equity among people living with chronic diseases.

## Resumen

**Objetivo:**

Analizar la investigación realizada por enfermería en intervenciones multinivel de automanejo con el fin de disminuir las disparidades de salud entre las personas que viven con enfermedades crónicas.

**Síntesis de contenido:**

Las intervenciones multinivel se han convertido en el núcleo de la investigación en enfermería en la última década. Sin embargo, una limitación crítica de las intervenciones existentes que se enfocan en las disparidades de salud entre quienes viven con enfermedades crónicas es la tendencia a abordar factores individuales o de nivel individual únicamente.

**Conclusiones:**

La investigación en enfermería está creando conocimiento que puede traducirse en la práctica clínica y promueve prácticas de autocuidado innovadoras y basadas en evidencia para disminuir las disparidades en la salud y promover la equidad en la salud entre las personas que viven con enfermedades crónicas.

## Resumo

**Objetivo:**

Analisar a pesquisa realizada pela enfermagem em intervenções multiníveis de autogestão para reduzir as disparidades de saúde entre pessoas que vivem com doenças crônicas.

**Síntese de conteúdo:**

As intervenções multiníveis tornaram-se o núcleo da pesquisa em enfermagem na última década. No entanto, uma limitação crítica das intervenções existentes que visam as disparidades de saúde entre aqueles que vivem com doenças crônicas é a tendência de abordar apenas fatores individuais ou de nível individual.

**Conclusões:**

A pesquisa em enfermagem está criando conhecimento que pode ser traduzido para a prática clínica e promovendo práticas de autocuidado inovadoras e baseadas em evidências para diminuir as disparidades de saúde e promover a equidade na saúde entre pessoas que vivem com doenças crônicas.

## Introduction

Changes in healthcare management have allowed more people to live with chronic diseases (CD) that were once acute and life-threatening.^1^ A CD is a physical or mental health condition that extends for more than one year leading to functional restrictions and increasing medical care needs.^2^ CD have become a significant U.S. public health issue, where 60% of adults live with one or more^3^ and 27.2% have multiple CD, representing over 85% of annual health care costs, or 3.8 trillion dollars.^1^^,^^2^ The consequences of CD significantly impact the social, mental, and physical health of people living with them. Usually, CD are reflected in poor quality of life and negative consequences for individuals, caregivers, families, and communities.^4^ Cancer, diabetes, hypertension, stroke, asthma, and HIV are examples of CD leading to hospitalization, long-term disability, and mortality.^4^

Diverse CD share common challenges associated with their management, including symptom recognition, medication adherence, and complex regimens.^5^ Individuals must also develop strategies to maintain proper nutrition, exercise, adjust to psychosocial demands (e.g., lifestyle regimens), and engage in effective relationships with healthcare providers over time.^5^ Therefore, self-management offers promise for a better understanding of the disease's symptoms and management. 

In addition, a CD self-management approach helps inform the development of strategies for evaluating health outcomes focused on decreasing health disparities and increasing the wellness of people facing CD.^6^^,^^7^ Health disparities usually result from social determinants of health (SDOH), or the conditions in places where people live, learn, work, and play, affecting a wide range of health-related outcomes and, consequently, individuals' quality of life.^8^. In the last decade, approaches to decreasing health disparities and improving people's quality of life with CD have increasingly become the core of nursing research.^1^^,^^9^

A critical limitation of existing interventions among those living with CD is the tendency to solely focus on single or individual-level factors. Multilevel intervention research are studies that include interventions targeted at various levels of the socio-ecological models and individual and biological factors closely related to individual health outcomes.^10^ This article aims to discuss multilevel self-management approaches in nursing to reduce health disparities among people living with CD.

### Self-management as a Science

Conceptual clarity regarding self-management and its integration into clinical practice is still a concern in the nursing community.^11^ As a general health concept, self-management is an individual's ability, in collaboration with their family, community, and healthcare providers, to manage symptoms, treatments, lifestyle changes, and the consequences of health conditions.^12^ Self-management also implies monitoring the illness and using cognitive, behavioral, and emotional strategies to maintain or manage health changes.^5^ A recent definition encompassing all the previous concepts describes self-management as an intrinsically controlled ability to live with the medical role and emotional consequences of CD in partnership with social networks and healthcare providers.^11^

Historically, nursing science has explored self-management for its impactful role in disease prevention, health promotion, and symptom management. For 30 years, The National Institute of Nursing Research (NINR) in the U.S. has been focused on supporting and promoting research into the development and broader application of self-management. The science of self-management supported by the NINR is based on the individuals' and families' responsibility as active participants in maintaining the well-being of those living with CD.^1^^,^^9^

Recently, with the leadership of the new NINR Director, new research priorities have been established.^9^ These new priorities include decreasing racism, using multilevel perspectives to implement interventions to address SDOH, and using nursing science approaches to advance precision health.^9^ This approach targets individuals, families, and the health system as a whole. Identifying upstream SDOH at the structural (e.g., policies, services, and environments) and individual levels (e.g., behaviors, epigenetics) are the hallmarks of this new paradigm. Moreover, this approach intends to identify all these factors to develop, test, and implement interventions to address SDOH involved in the CD self-management process, consequently contributing to decreasing health disparities among individuals with CD.^9^

### Health Disparities and its Relationship with Self-management and Multilevel Intervention Research

Health disparities are a difference in the outcome or the incidence/prevalence of disease, earlier onset or faster progression, poorer daily functioning or quality of life, mortality, and burden of certain diseases and other adverse health conditions among disadvantaged groups.^8^ Health disparities can be further pronounced when characterized by race/ethnicity, income, insurance status, education, occupation, and other social factors.^8^ Although health disparities are manifested at the individual level, other contexts, such as community and public policies, should be considered when investigating the associations between disparities and clinical outcomes. 

Recognizing SDOH is the first step to combating health disparities. SDOH impact self-efficacy, health literacy, social support, health beliefs, motivation, and coping, which are relevant antecedents of self-management.^11^ Altogether, these factors impact healthcare and education access, the social environment, and health outcomes, affecting individuals’ ability with CD to self-manage their condition. Therefore, a holistic approach to self-management and health disparities should have an integral perspective and include interventions that address these factors together.

The focus of multilevel intervention research is to reduce the health disparities for affected populations.^6^^,^^7^ Therefore, multilevel interventions target the causes of health disparities by focusing on different levels of influence that affect health, including individual (intrapersonal), microsystem (interpersonal), mesosystem (institutional), exosystem (community), and macrosystem (public policy).^13^ According to Agurs-Collins and colleagues, multilevel intervention research requires action targeting at least two or more levels of influence simultaneously or in close temporal proximity.^14^ However, the approaches implemented at each level may vary in type and interaction with other levels through synergistic effects.^6^

For instance, multilevel interventions for self-management among people living with CD can be based on the socio-ecological approach. The model shows that interventions targeting factors at the individual level, such as education related to medication adherence, can be facilitated by upstream factors such as peer educators (interpersonal level) and social media campaigns to impact CD prevention (community level). At the institutional level, increasing testing sites (e.g., for diabetes mellitus, HIV, and hypertension) and promoting changes in the delivery of health services, including follow-up visits for high-risk populations, are effective options to prevent and manage typical CDs. The public policy level may include policy interventions, such as decreasing medication-related costs and increasing access to early diagnosis and treatment. This example covers potential intervention strategies that are complex and beyond the scope of a one-level intervention. In addition, it illustrates possible factors within and between levels that could be addressed through multilevel interventions to reduce health disparities and facilitate CD self-management. 

### Challenges and Opportunities of Multilevel Intervention Nursing Research in CD Self-management

Nurses have a leading role in proposing innovative strategies and developing training opportunities for researchers focused on CD self-management multilevel intervention studies (Table 1). Training programs on research methodology and multimethod approaches are potential areas of improvement in multilevel interventions.^14^^,^^15^ As such interventions address multiple SDOH, they involve intricate levels of design, analysis, implementation, and evaluation.^14^^,^^15^

**Table 1:** Training Opportunities to Promote Multilevel Nursing Research Studies on CD Self-management

Stage	Training Opportunities
Design and framework	- Novel and systemic theoretical frameworks addressing multiple levels
	- Integration of the multimethod approaches, including quantitative, qualitative, and mix-method research
	- Community-based participatory research
	- New metrics incorporating elements from theoretical frameworks to evaluate multilevel relationships.
	- Effectiveness of multilevel research designs to assess mechanisms associated with the self-management of chronic diseases.
Statistical analysis and approaches	- Research methodology and statistical techniques available, including advanced analytical tools (e.g., structural equation modeling, multivariable logistic regression models)
	- Convergence of different research designs
	- Analysis of the variables to understand causal pathways and mechanisms affecting self-management of chronic diseases
	- Analysis of the temporal effects of policies in the socio-ecological context and their impact on self-management of chronic diseases
Implementation and evaluation	- Reporting the context of research studies: transparent reporting of setting, site, and clinician selection
	- Reporting the integration of theory, models, and interventions
	- Measures for outcomes at different levels
	- Use of transdisciplinary teams and community involvement
	- Assess health care system policies and practices to incentivize and promote a multilevel approach

*Note.* Table developed by the researchers based on Agurs-Collins *et al*.,^14^ Paskett *et al*.,^7^ and Stange *et al*.^15^

Nurse researchers have begun to address these challenges and demonstrate the importance of this type of research and have identified a substantial need for additional methodological development to advance the field of health disparities research surrounding self-management of CD.^14^ In this context, using theoretical frameworks to guide multilevel studies is fundamental.^14^ The selection of an appropriate framework will support selecting appropriate measures and culturally appropriate interventions. One strategy is to develop interventions based on socio-ecological and bio-behavioral frameworks to identify the mechanisms linking SDOH of CD and health-related outcomes.^9^

Another challenge is the adequate selection of methodological approaches to address multilevel intervention research. It would be crucial to provide the resources and strategies to train more nurses on self-management and on utilizing the appropriate statistical techniques available, including the convergence of different research designs.^7^ Weak analytic plans, inadequate sample sizes, and statistical approaches that do not account for the complexity of data across levels are recognized as limitations when designing multilevel intervention studies.^14^ Further, strengthening innovative methods through the implementation of expert-led review panels, utilization of common data elements such as standardized data collection tools, and data collection in large populations are strategies for designing multilevel intervention studies.^1^

The lack of transdisciplinary teams needed to design and evaluate multilevel interventions and the lack of sufficient time and resources are critical challenges to consider.^14^ Multilevel interventions require significant monetary effort for which researchers and potential grants must be prepared. Multilevel intervention designs must also consider the time frame, which is usually more extensive than single-level interventions, and the larger sample sizes with extended follow-up periods that are often required to see synergistic effects between and within levels.^15^

The use of multimethod approaches that integrate quantitative and qualitative research across multiple levels is essential for outcomes of interest in health disparities research.^15^ Simultaneous data collection using both approaches may provide opportunities to work with small groups and identify specific interlevel processes. It is relevant to mention that community-based participatory research and implementation science approaches can also provide opportunities to execute successful multilevel research among people living with CD.^9^

Statistical challenges for multilevel studies include analyzing outcomes at each targeted level and examining mediators and moderators involved in these relationships.^6^^,^^16^ Nurse researchers should be cautious about the lack of independence between the variables due to the correlation or clustering of data. Robust research methodology and available techniques' training on advanced analytical approaches and power assessment are necessary to evaluate outcomes.^6^^,^^16^

A relevant challenge faced by researchers during the implementation of multilevel research is the historical lack of engagement at the public policy level. Vital SDOH disparities often lie within upstream ecological levels.^14^ Achieving long-lasting improvements to individuals and communities will likely require buy-in from politicians and others at the policy level.^7^ Adding public policy-level changes to the interventions might result in greater proximal and distal outcomes. 

Unfortunately, few detailed reports have described how multilevel interventions have been implemented in a successful way.^15^ Although multilevel interventions are contextual, most reports fail to report the studies' process adequately.^15^ This situation can result from uncontrolled and unpredictable changes in contextual variables (e.g., transportation, access to medical care) within and across levels.^14^ Such changes may be more remarkable among people living with CD because these intervention-relevant factors can be less stable and change over time.^14^ Consequently, a more detailed report of the setting, site, researchers, context and range of applications is needed.^15^

Further, training grant reviewers who focus on assessing the accuracy of the setting for this approach and on the empirical evaluation of interventions is critical. Parameters such as fidelity or acceptability can be challenging to maintain in low-resource settings with competing health and welfare priorities.^14^ Remarkably, the National Institutes of Health in the U.S. emphasizes the need for researchers to describe the feasibility, generalizability, acceptability, sustainability, and accessibility of research findings of available interventions, especially for underserved and vulnerable populations.^9^

## Conclusion

Self-management is a complex phenomenon that implies intersectoral work to target health disparities among disadvantaged groups, especially those living with CD. Given that a broad range of factors make up the CD self-management process, nursing research utilizing multilevel interventions is needed. Despite challenging concerns, multilevel intervention studies may be most effective at reducing health disparities, having a broader public health impact among underserved populations than interventions focusing on only one or two levels. These may help not only to prevent CD but also to encourage and inform individuals on effectively self-managing their CD.
